# Male adolescents’ role in pregnancy prevention and unintended pregnancy in rural Victoria: health care Professional’s and educators’ perspectives

**DOI:** 10.1186/s12884-018-1886-y

**Published:** 2018-06-18

**Authors:** S. Connor, K. Edvardsson, E. Spelten

**Affiliations:** 1 Department of Rural Nursing & Midwifery, La Trobe Rural Health School, Vic, Mildura, 3500 Australia; 2 Judith Lumley Centre, School of Nursing and Midwifery, La Trobe University, Vic, Bundoora, 3086 Australia; 3 Department of Clinical Sciences, Obstetrics and Gynecology, Umeå University, 901 87 Umeå, SE Sweden; 4 Department of Public Health, La Trobe Rural Health School, Mildura, Vic 3500 Australia

**Keywords:** Rural adolescent males, Health care providers, Educators, Teenage pregnancy, Qualitative study

## Abstract

**Background:**

While there has been a steady decline in adolescent pregnancies worldwide and in Australia over the last three decades, Australian rates still rank third highest among developed countries. Adolescent pregnancies are defined as those that occur to girls between the ages of 15 and 19. The current pregnancy rate of 15 to 19 year old females rural Victoria is 21.19%, this is more than double the Victorian state rate of 8.2% and almost double the national Australian rate at 13.1% The aim of this study was to explore Health Care Professionals and Educator perspectives on these high adolescent pregnancy rates, with particular focus on the role of adolescent males, in a rural region in Victoria, Australia.

**Methods:**

A qualitative descriptive study using focus group discussion was undertaken with Health Care Providers and Educators (*N* = 8) in 2016. Data was analysed using thematic analysis.

**Results:**

Four themes emerged from analysis. The first, ‘Gender Stereotyping’ focused on the acceptance of traditional masculinities; the second ‘Adolescent males as health consumers’ was based on the consensus that adolescent males are poor consumers of health and ‘invisible’; the third ‘Complexity of Issues’ identified that, particularly in a rural region, contributing issues are varied and complex; and the fourth ‘Focus on Fatherhood’, saw the participants diverge from the discussion about pregnancy prevention and the adolescent males’ role in unintended pregnancy, and focus on the role adolescent males may have as unintended fathers.

**Conclusions:**

Participants did not consider young males to be of importance in the prevention of adolescent pregnancy. There is a need to further explore the role of young males in pregnancy prevention, including what role traditional gender stereotyping, from health professionals’ and young males’ perspectives, plays in provision of adolescent sexual health services.

## Background

Despite the worldwide decline in adolescent pregnancies, rural adolescent teen pregnancy rates continue to rise in Australia. Adolescent pregnancies are defined as those that occur to girls between the ages of 15 and 19 and while there has been a steady decline in adolescent pregnancies and births worldwide since 1990, currently 11% of all births are to girls aged 15–19, and 95% of these births occur in low and middle-income countries [1]. In Australia adolescent pregnancy, rates have also declined but in rural Victoria, the current pregnancy rate for 15 to 19 year old females is 21.19%. This is more than double the Victorian state rate of 8.2% and almost double the national Australian rate at 13.1% [2].

While not all adolescent pregnancies and births are unplanned, the often negative consequences of being an adolescent single parent and the effects for the children and society, are well documented. Among the most significant consequences for adolescent single mothers and their children, are an increase in welfare dependency and poverty. Young mothers are also at high risk of poor emotional health including depression and anxiety, which may be linked to social isolation due to disengaging from high school and becoming isolated from peers [3, 4]. Non-completion of secondary schooling reduces employment opportunities [3, 4]. Children of adolescent mothers are also at higher risk of developing behavioural problems, being injured in an accident, and having lower levels of literacy and numeracy skills [1, 3–5].

The focus of this study is adolescent males in a rural Victoria region. The particular region was identified for several reasons. The region has a population of approximately 59,000 and includes an indigenous population of 2516 people, a further 30.7% of total population were born overseas [6]. The region has been identified as the third most disadvantaged region in Victoria, with only 22.9% of residents completing Year 12 level schooling and an unemployment rate at 6.6% compared with the Victorian state average of 5.4% [7].

With a rate of adolescent pregnancy at over half the national average, this region has a significant number of adolescent females and males who experience unintentional pregnancy. The purpose of this study was to explore Health Care Professionals’ and Educators’ perspectives on prevention of adolescent pregnancy and the male adolescents’ role in unintended pregnancy and pregnancy prevention, as a growing body of evidence suggests that Health Care Professionals are failing to provide adolescent males with support services, equivalent to those, that are made available to young adolescent women [8–12].

## Methods

### Research design

This study utilised qualitative descriptive methodology. Qualitative description is a paradigm of qualitative research that requires the researcher to provide a detailed interpretation of the data that is explained in every day terms rather than through a theoretical lens. Qualitative descriptive studies draw on naturalistic inquiry to enable the researcher to establish solid and meaningful findings [13–16].

Purposive sampling was used to recruit participants, and inclusion criteria for participants included being a Health Care Provider or Educator currently working in the identified rural Victorian region. There was no pre-requisite regarding experience with either adolescent pregnancy or male adolescent sexual health.

Twenty-three potential participants were contacted via email and provided with written information about the study, aim and procedures, eight of whom took part in the focus group. Three potential participants responded but declined for various reasons including work and family commitments. Eight potential participants did not respond to the invitation or the two email reminders. Twelve potential participants in total accepted the invitation; however, four did not attend on the day. An overview of the participants is presented in Table 1.

**Table 1:** Participant overview

Midwives	2
Social worker	3
Sexual health nurse	1
Mental health nurse	1
GP	1
Females	6
Males	2
Education sector	1
Health care sector	7

A focus group discussion was chosen as the best method for data collection as this forum allows homogenous groups to come together to explore views, feelings and experiences regarding a specific topic for the purpose of collecting research data [17–20].

Five questions were developed that were intended to provoke in-depth discussion amongst the group members. The participants were asked to consider and share their experiences with adolescent males that they may have had in regard to pregnancy prevention and unintended pregnancy, in their role as professional caregivers and educators. To aid in the generation of meaningful data, further question were then posed regarding the participants knowledge of current health services for adolescent males, and any challenges they were aware of in this area. Finally the participants were offered the opportunity to add a final comment to the discussion.

The discussion was moderated by the first author (SC) and was recorded for verbatim transcription. Field notes were taken during the discussion to add richness to the data set by both co-authors (KE & ES). All participants were provided with a pseudonym, de-identified transcripts were returned to the participants for comment and member checking, prior to commencing analysis.

Thematic analysis was determined as a suitable approach to use with qualitative descriptive research. It is a rigorous systematic framework used to identify themes within the collected data, that lead to an understanding of the participants’ experience [14–16, 21]. Analysis commenced with the familiarisation of the transcribed Focus Group discussion and field notes, by first author, SC. All data was read repeatedly and patterns and meanings, both manifest and latent, were actively looked for during this phase [22]. Once familiarity with the data was achieved, a list of initial codes was produced. Each code included relevant content from the data [16]. The codes where then clustered into potential categories using colour codes to identify the category with the code and data. These categories were reviewed and collated into larger themes, ensuring that the data included within each was both distinct and meaningful. A thematic map was developed at this stage to help describe the relationship between the codes, categories and the themes [16, 22]. The thematic map and coded data sets were then shared with the co-authors, ES and KE, for further review. A detailed analysis of each theme was conducted ensuring that each theme was authentic and congruent with the overall narrative [16, 22].

The consolidated criteria for reporting qualitative research (COREQ) checklist, was utilised to determine rigor of research design and transparency of reporting of results [23].

## Results

Analysis of the focus group transcripts provided four clear themes, emerging from the categories and codes. The following discussion will provide a description of the themes using quotations from the focus group to illustrate.

### Gender stereotyping

The first focus group question ‘what is your experience of adolescent males in relation to pregnancy and pregnancy prevention’ was posed as a way of initiating conversation around adolescent males within the participant group. The participants responses clearly indicated themes relating to gender stereotyping (Table 2).

**Table 2:** Gender stereotyping

	Codes	Categories	Theme
• Adolescent boys are not seen in Health care services • They don’t often seek help/support at school • We don’t consider the needs of boys in our service • I’ve never thought about teenage pregnancy from the male perspective	Invisibility	Masculinity	Gender stereotyping
• Young boys sow their wild oats • They refuse to wear condoms • They wouldn’t take a male pill if it was available • Boys have no responsibility	Boys will be boys		
• Young girls want to become pregnant • Once she is pregnant it is her choice -not his • The female has the right to choose	Her body -Her choice	Femininity	
• I only see the female once she is pregnant • The female is my patient/client not her boyfriend • The focus of care is on the mother to be	She is the client		

The initial response from all participants was a strong feeling that ‘boys will be boys’

*“They’re quite proud of spreading their seed around and making lots of babies and not sticking around with the mums, where I am from”*

Participant B, Sexual-Health Nurse.

However, this initial response quickly faded away to be replaced with a real sense that adolescent males are rarely seen by Health Professionals or Educators in relation to sexual health and adolescent pregnancies. Several participants expressed this opinion quite strongly,

*“I would say where I work, with the community that I work with, they are almost invisible.”* Participant C, Community Midwife

*“I have very few health encounters where an adolescent male will come in to talk about pregnancy or pregnancy prevention.”*

Participant E, General Practitioner.

The discussion regarding the male adolescent role was very limited. Moreover, perhaps can be best summed up by one participant, who stated,

*“This [adolescent pregnancy] wasn’t something that I had ever considered from the male perspective.”*

Participant H, Primary Mental Health.

The participants however did express more detailed views regarding how they saw the female in this situation. The two main schools of thought regarding the young females, related to what they considered ‘feminist’ issues and their own professional standards. The participants felt that young women had to some degree taken on board a feminist stance and in doing so perhaps prevented the male’s right to contribute to a decision.

*“You know where it’s [continuing with the pregnancy] considered a female decision.”*

Participant F, Social worker [Education]

*“[the girls say] this is my body my rights.”*

Participant A, Social worker.

One of the strongest views to arise at this time however was around professionalism. The health professionals in particular were quite adamant that the female, whether seeking pregnancy prevention advise or care during pregnancy, was their client and the male partner was not. Therefore, the focus for them was on the female.

*“When we had a young person that was pregnant all the focus would be about getting the female to appointments and putting all the supports around her.”*

Participant F, Social worker

*“…and, well they’re [the male] not your client per se.”*

Participant C, Community Midwife

*Well I can only speak from a medical professional, I’ve no right to see their [the males] medical history. I’m her carer so unless there is a reason to know or he chooses to tell me I don’t have any real need.*

Participant E, General Practitioner.

### Adolescent males as health consumers

Overwhelmingly all participants agreed that adolescent males were difficult to engage in any form of health initiatives let alone sexual health and pregnancy prevention (Table 3). Unlike adolescent females who need to present to a sexual health service or GP clinic for contraception or health checks, there is limited need for an adolescent male to seek health advice.

*“You don’t need a script for condom you know, so, that’s taken out of the face to face contact, but, so you miss that opportunity to have that discussion.”*

Participant E, General Practitioner

**Table 3:** Adolescent males as health consumers

	Codes	Categories	Theme
• Services at High Schools with Mental Health Focus • Sexuality Education focused on females	Mental Health & Wellbeing	High School Services	Adolescent Males as health Consumers
• Boys are lost after primary school • No incentive for boys to seek health care	Disengagement	Sex Education	
• Religion has an influence on sex education in private schools	Religion		
• Focus on STIs not pregnancy • Males respond more to STIs as they are affected • STIs are treatable so they (boys) don’t care	Boys & STIs		
• Males only seek health care in emergencies • Males rarely engage with our sexual health service • We only see them if they are dragged in by their partner • It’s difficult enough to engage adult males let alone adolescents	Getting males to see a HP	Accessing Health	
• There is a need for male sexual health workers	Male health Workers

*“Occasionally I’ll see a boy, a male, in that age group if he actually has some weird something coming out of his penis. Otherwise we rarely see them…”*

Participant B, Sexual-Health Nurse.

All the participants were able to discuss a wide range of services that they knew of that were available to young adolescent males in the region. These services however were in the whole, related more to Mental Health and Wellbeing than sexual health. There was only one dedicated sexual health service represented and they admitted to not seeing many young males.

*“We rarely get boys in unless they are either dragged in by their partner or they have actually got symptoms that worry them.”*

Participant B, Sexual-Health Nurse.

This prompted discussion around adolescent males and Sexually Transmitted Infections (STIs), most participants felt that targeting the young male’s health from this perspective might be more successful than trying to engage them in pregnancy prevention. STI’s present and actual immediate impact to their lifestyle.

*I guess talking about the use of condoms and things, and not just saying that it prevents pregnancy but also sexual disease. You know we keep saying um, you need condoms to prevent pregnancy but what about the sexual health.*

Participant D, Community Midwife

*“It may actually be generally a better approach. Men may actually respond to that because there is an outcome that they don’t want.”*

Participant A, Social worker.

Once the participants had identified this lack of need for adolescent males to seek health care, they linked this back to Sex Education in schools. The participants felt that this was the last chance they had to interact with young boys before they left primary school and entered adolescence. Those services that provided Sex Education did so to either the grade five or six class, making the children around 10–12 years of age. There were several different programs depending on state and school. One service had begun an ‘Advanced Sex Ed’ class, which included the parents and contained some explicit information regarding consent, pornography and body image.

*Just going that one step further about consent and um, a little bit more a bit about, bit about pornography and um, body image and that sort of stuff and stepped it up just that little bit further into talking about sex really.*

Participant B, Sexual-Health Nurse.

School based sex education however appears to be ad-hoc at best. One health care professional expressed her frustration at needing to be ‘slotted’ in amongst the school’s other curriculum requirements.

*It’s very difficult these days to actually get into the schools, they plan their curriculum the year before if not two years before um and unless you factor that in.*

Participant B, Sexual Health Nurse.

There is also the issue of the type and quality of sex education that is being provided through school-based programs. In particular, the parochial schools in the region do not participate in nurse led sex education programs. There was concern amongst the focus group participants that this may lead to the students being taught sex education based on the tenants of their religion rather than evidenced based best practice.

*I did talk to the, one of the Vice Principal’s there [Parochial School] about what they do in terms of contraception education, he said they talk about everything except termination and the morning after pill because they are abortion.*

Participant B, Sexual Health Nurse.

However, the group consensus was that school based sex education programs often felt futile, as this was often the last contact that that any health service has with the males and they become disengaged from this point until well into adulthood.

*“We have identified that they get lost once they go to high school.”*

Participant B, Sexual-Health Nurse

*“Men’s health in general is difficult, it’s hard to get men engaged generally so young men who just don’t care about anything apart from themselves really is even more difficult.”*

Participant B, Sexual-Health Nurse.

All participants of the focus group bar two were female and this was an accurate representation of the regions workforce. The participants all felt that one area that may go a long way to improve adolescent male health interactions was in developing a more gender balanced health work force within the health care and education sectors.

*I think it’s a disadvantage being a female, when we had a young male health worker we seemed to get more young men in but they don’t want to see, someone the age of their mother*

Participant B, Sexual-Health Nurse

*“I just think if there were more perhaps male nurses doing sexual health, we would have better access to young males”.*

Participant C, Community Midwife.

### Complexity of issues

All participants agreed that the issue of adolescent males and their sexual health needs was a complex issue involving, but not confined to, risk taking behaviors such as alcohol and illicit substance abuse. The issue is complicated further by the rural location and the implication of low socio-economic status, limited access to sexual health services and a real or perceived lack of confidentiality within a small community. These themes came up repeatedly throughout the focus group discussion (Table 4).

**Table 4:** Complexity of issues

	Codes	Categories	Theme
• Perceived & real lack of confidentiality • Family or friends work in health services • Local issues with confidentiality	Confidentiality	Rurality	Complexity of Issues
• Not enough services for adolescents in general • Limited access to choices such as termination • There is a need to travel to many services	Access		
• Parents of adolescents often uneducated • Adolescent motherhood is a generational issue • There are low expectations for adolescents in this region • Many don’t finish school	Socio-Economic Status		
• Alcohol and drugs are often involved • Alcohol and drugs lead to poor choices	Drugs/Alcohol	Risk Taking Behaviours	
• The boys just don’t care • It can be seen as ‘cool’ to be a young parent	Ambivalence		
• Its normal for indigenous community • There are cultural issues involved • Our indigenous boys’ health checks are not focused on sexuality	Cultural	Indigenous	

A noteworthy consideration at this point, is that, while the participants all acknowledged adolescent males as the more likely risk takers, the focus of their discussion continually returned to their experiences with adolescent females.

Risk taking was seen by the participants to be a generally accepted adolescent behavior, which led to poor decision-making followed by ambivalence of the consequences. In particular they had concerns regarding use of drugs and alcohol in the community for both genders, but were only able to relate instances regarding adolescent females.

*“Yeah I think systemically we have got significant issues with risk taking behaviours like drugs and alcohol”.*

Participant F, Social worker [Education]

*Somebody who comes in and says. ‘oh, I would never do that, or I would never consider myself to be someone who is promiscuous but, it was the drugs and the alcohol*

Participant E, General Practitioner

*One of the things that we haven’t really talked about in this particular question is around drugs, in particular, and the impact on choices that they {females] make with regard to their sexual behaviour and I think it’s really disturbing, frankly, you start being substance affected and you don’t care.*

Participant A, Social worker.

The participants also felt that adolescents of both gender where ambivalent regarding the outcomes of unprotected sex.

*I think there’s ambivalence on both parts though, like from what I see they’re pregnant, they’re not actually wrapt about it, but they’re not too bothered that’s just what happened, go on with it, and he kind of just, is the same um probably there is less expectation that he will be involved but, everybody seems reasonably okay with that scenario, is what I kind of see, yeah*

Participant C, Community Midwife

All participants agreed that the regions geography played an important part in the high statistics for unintended adolescent pregnancy as does, a lack of access to family planning services and a perceived lack of confidentiality amid the services that are available in a small community However they were unable to discuss this from the adolescent male perspective and focused instead on the adolescent female who was unintentionally pregnant.

*To get a termination in [this region] you’ve got to go see your GP by 13 weeks you can’t just get on the train and go to Melbourne whereas if your somewhere in a town closer to Melbourne it’s not as difficult to get a termination*

Participant C, Community Midwife.

This was the same for the discussion around socio –economic status. It was generally agreed that one of the major reasons for the high adolescent pregnancy rates was directly related to the low socio-economic status of the region. However the focus for this discussion was limited to the population in general rather than the adolescent males.

*“I also do think that the population that we have and the demographics that we have has a big bearing on the outcomes so, the high teenage pregnancy rates”.*

Participant D, Community Midwife

*The socio-demographics really need to be looked at, and education, possibilities, options, opportunities that are in [the region], and how its [education] valued here or not valued here, and the percentage of parents who are not employed.*

Participant A, Social Worker.

One smaller sub-theme, adolescent Indigenous health, emerged in this category; it is limited to a sub-theme, as it did not generate a significant amount of data. However, considering the rural location of the region and the large indigenous population, as previously outlined, it warrants inclusion. Only one participant, differentiated between indigenous and non-indigenous adolescents.

*We also have the highest aboriginal population and it its normal for aboriginal girls to start their families younger. We have the highest per capita aboriginal population in the state so.*

Participant C, community Midwife

The research team did not separate indigenous adolescent males from the research question cohort as, at this stage, the study is exploring Health Care providers and educators’ perspectives on adolescent males in general, not specific groups of the population.

There was considerable knowledge amongst the focus group participants regarding various Health Education Programs aimed at Adolescent Indigenous Males, though none of these has a focus on Sexual Health or Pregnancy prevention.

### Focus on fatherhood

One of the strongest themes to emerge from the data was Focus on Fatherhood (Table 5). It was evident from previous themes that consideration of adolescent males in relation to pregnancy prevention and unintended pregnancy was a new perspective for the focus group participants. Many of them though had strong views on adolescent males as Fathers. There was a general group consensus that adolescent males are not willing to commit to parenting. A perception however, that was not necessarily based on clinical experience but personal opinion.

*and I think they [adolescent males] start off quite positive like with good intentions, but then they don’t know how to make it work possibly cause all the other stuff gets in the way you know whether it be poverty or drugs.*

**Table 5:** Focus on fatherhood

	Codes	Categories	Theme
• Young men want to be fathers too • We need to support our young dads • We need to teach them how to be dads	Support them as fathers	Fatherhood	Focus on Fatherhood
• Young mums can be at risk of domestic violence • Many young couples have complex legal issues • We need to protect the young mothers	Violence	Partners needs	
• Adolescent males do not commit to parenting • Successful teen couples are a minority	Young males don’t commit		

Participant F, Social Worker [Education]

***“*** *Well they turn 18 and they’ve got freedom”.*

Participant C, Community Midwife

***“*** *Who wants to be home with the baby when they could be out partying with their mates”.*

Participant B, Sexual-Health Nurse

Several of the participants whose services provided assistance for adolescent mothers expressed that often exclusion of the male partner was due to their concern for the safety of the young women in their care.

*The relationships are so volatile aren’t they, they’re in love one minute a week later and they’re in court getting an intervention order and then you know they’ve moved on to the next partner.*

Participant F, Social Worker [Education]

*The cycle of violence is, such as it is, I’m assuming everyone understands probably better than I do and um you know that’s another reason why we are pretty protective around the mums.*

Participant A, Social Worker.

The final discussion point relating to adolescent males as fathers saw the group participants recognise that there is very limited support available to any adolescent males who do intend to stay involved in the pregnancy and take on the role of fatherhood.

*You [females] go to your maternal and child health nurse and you learn you know certain techniques and things you can do to settle, but the guys don’t learn that, so they need to be included in that process as well, so they can feel confident because I think a lot of it is also confidence.*

Participant G, Social Worker

*Even to the point where um men can get, I think its two weeks paid leave that’s it. You know we don’t as a society or a government or whatever value the male’s role in that parenting anyway, so you know it’s from the top down really.*

Participant C, Community Midwife

*It’s also about the need for organisational and cultural change, so as service providers, we’re thinking about adolescents as parents, adolescent males as parents rather than the focus that is on the females all the time. As service providers we actually start that conversation to get it more mainstream.*

Participant D, Community Midwife.

## Discussion

This purpose of this study was to explore Health Care Professionals and Educators perspectives on the male adolescents’ role in unintended pregnancy and pregnancy prevention in a rural Victorian region.

There are several limitations to this study, including the small sample size, with only eight participants in the focus group. However the authors believe that the participants were a fair representation of the health care professionals within this rural community and quality data was obtained. Another limitation is that whilst one Health Care Professional was working in the education sector, no educators attended. This in itself it not a considerable issue as all Health Care Professionals act in the role of educators when providing client care. Furthermore, six of the eight participants were female and only two males. While this is representative of the gender balance within the health sector, having more males involved in the focus group may have given the data broader gender perspectives.

While it is self-evident that adolescent males are an important link in preventing unintended pregnancies, the findings of this focus group discussion clearly indicated that, health care professionals and educators in this rural region, do not consider them to be relevant in this context and that their focus is neither on male adolescents nor on prevention but mostly on supporting the adolescent mothers with unintended pregnancies.

There was a strong perception amongst the focus group participants that despite current discourse around gender equity, traditional gender roles play a role in the increasing number of unintended adolescent pregnancies. Traditional masculinity socialises young men to project strength and dominance, particularly over women [24, 25]. The focus group participants observed that the behaviour of the adolescent males in this rural region was typically ‘boys being boys’ who were ‘sowing their wild oats’. Not only does this traditional masculinity seem to be accepted as normal in this rural region but the focus group participants did not believe that it was within their power to positively impact on this ideology. The young females in contrast are seen to be control of their own choices and bodies. The focus of care provision for the participants is with the adolescent females who are unintentionally pregnant.

Much of the published literature refers to adolescent males as invisible and hard to reach [8–10, 26]. The findings of this research project appear to substantiate this data. The health care professionals and educators involved in the focus group discussion, all identified adolescent males as a cohort that is very difficult to engage in any kind of health care strategy let alone one that is focused on pregnancy prevention and unintended pregnancy. This lack of interaction with health care, correlates with traditional masculinity norms that reinforce beliefs around the male body being strong [24, 25]. In contrast to this, the same participants were not consciously aware prior to participating, that adolescent males were not present within the context of the adolescent unintended pregnancies they came into contact with. This raises questions as to how health care professionals and educators can change their practices to include adolescent males in the care of their partners when experiencing unintended pregnancies.

The issue of adolescent males, pregnancy prevention and unintended pregnancy is clearly complex. The focus group participants identified a multitude of contributing factors, some such as confidentiality and access to family planning services, are related to the rurality of the region [27]. Others such as, male risk-taking behaviours and ambivalence, are further related to traditional masculinity [24, 27]. While all participants agreed strongly that these factors were real and important, they felt disempowered to address these issues in any significant way. The participants also had difficulty considering these issues from the adolescent male perspective. With the exception of risk –taking behaviours, all issues were only discussed from the perspective of the adolescent female who is unintentionally pregnant. This again serves to illustrate the limited contact with adolescent males that the participants have.

A significant positive change the focus group participants discussed, was potentially increasing support for adolescent males who unexpectedly found themselves as prospective fathers. Some participants still held reservations around the apparent suitability of the adolescent males to be involved with the mother and child, citing cycles of violence and lack of commitment on the males’ part as worrying issues. Interestingly both violence towards partners and lack of commitment are seen as components of traditional masculinity, which once again reinforces the accepted stereotype in this rural region [24, 27]. The greater majority of participants however felt that benefits could be gained for all concerned if they were able to provide greater level of support to adolescent males who choose to remain involved in the pregnancy and play a role in fatherhood. This ideal is supported by Bunting & McCauley [8], Osborn [9] and Paranjothy et al. [10], who all identified that many young fathers, rather than being invisible and hard to reach, actually feel excluded from being involved in the pregnancy and fatherhood by Health Care Professionals.

## Conclusion

In order to prevent and reduce unintended adolescent pregnancy, health care professionals and educators need to acknowledge that adolescent males are an integral part of this complex issue. Acceptance of traditional masculinity behaviours as an explanation for perceived adolescent male behaviour only serves to perpetuate gender stereotypes and does not help to solve the problem.

Further research to gain an understanding of adolescent males’ perceptions of their own masculinity may be helpful in providing health care workers and educators enhanced understanding of the decisions that these young men make and work towards dispelling the gender stereotypes.

The responsibility for finding these invisible and hard to reach young men, rests in the professional duty of the health care professionals and educators. These professionals need to alter their current thinking and begin to develop more inclusive practices so that the significant issue of prevention of adolescent unintended pregnancy can be addressed.

## Abbreviations

STI’s: Sexually transmitted infections
